# Diagnostic Accuracy of Biomarkers for Early-Onset Neonatal Bacterial Infections: Evaluation of Serum Procalcitonin Reference Curves

**DOI:** 10.3390/diagnostics10100839

**Published:** 2020-10-18

**Authors:** Hidetoshi Go, Nobuhiko Nagano, Daichi Katayama, Takuya Akimoto, Takayuki Imaizumi, Ryoji Aoki, Midori Hijikata, Ayako Seimiya, Ryota Kato, Aya Okahashi, Ichiro Morioka

**Affiliations:** Department of Pediatrics and Child Health, Nihon University School of Medicine, Tokyo 173-8610, Japan; go.hidetoshi@nihon-u.ac.jp (H.G.); nagano.nobuhiko@nihon-u.ac.jp (N.N.); katayama.daichi@nihon-u.ac.jp (D.K.); akimoto.takuya@nihon-u.ac.jp (T.A.); imaizumi.takayuki@nihon-u.ac.jp (T.I.); ryoji_kamo@yahoo.co.jp (R.A.); hijikata.midori@nihon-u.ac.jp (M.H.); seimiya.ayako78@nihon-u.ac.jp (A.S.); kato.ryota@nihon-u.ac.jp (R.K.); okahashi.aya@nihon-u.ac.jp (A.O.)

**Keywords:** C-reactive protein, immunoglobulin M, sensitivity, sepsis, specificity, white blood cell, Youden index

## Abstract

To date, no clinical studies have compared the accuracy of serum procalcitonin (PCT) reference curves. We aimed to validate the diagnostic accuracy of previously reported serum PCT reference curves and to determine which biomarkers among a cut-off value over the 95th percentile in the serum PCT reference curve, white blood cell (WBC) count, and C-reactive protein (CRP) and immunoglobulin M (IgM) levels, have the highest diagnostic accuracy for early-onset neonatal bacterial infections. This retrospective cohort study assessed 16 preterm and 23 term infants with suspected bacterial infections within 72 h after birth. Each infant group was divided into two subgroups: confirmed- and non-infection. The diagnostic accuracy was determined using the Youden index. The reference curves by Fukuzumi et al. in preterm and term infants had the highest Youden indexes: 1.000 and 0.324, respectively. Among preterm infants, the Youden index for PCT was 1.000. Among term infants, the Youden index for a combination of PCT, CRP, and WBC and/or IgM was 1.000. In conclusion, a serum PCT level over the 95th percentile on the reference curve for preterm infants and a combination of PCT and CRP levels with WBC count and/or IgM levels for term infants provided sufficient diagnostic accuracy.

## 1. Introduction

Early diagnosis and treatment of early-onset bacterial infections in neonates are important because they have a high mortality rate and result in serious sequelae [1,2,3]. The white blood cell (WBC) count and serum C-reactive protein (CRP), immunoglobulin M (IgM), and procalcitonin (PCT) levels are commonly used as biomarkers for early-onset neonatal bacterial infections in clinical settings worldwide [4,5,6,7].

CRP is synthesized mainly in the liver during the acute phase of a bacterial infection. However, the CRP level is also elevated in the presence of other diseases, such as collagen diseases, malignancies, and asphyxia, other than infectious diseases [8,9]. The WBC count is well known to be increased in infections, especially bacterial infections. Rodwell et al. have proposed a hematologic scoring system using the WBC count, polymorphonuclear leukocyte count, and immature to total neutrophils ratio for the diagnosis of neonatal sepsis [10]. An elevated IgM level in neonates at birth is a biomarker of early-onset bacterial infections, as IgM does not cross the placenta to the fetus [7,11]. In particular, the combination of CRP and IgM has been reported to be a more accurate diagnostic marker for the detection of neonatal bacterial infections [7,11].

PCT is a precursor of calcitonin. It is produced and secreted by thyroid C cells in the normal physiological state, but proinflammatory cytokines in response to bacterial organisms and toxins act primarily on the liver to promote PCT production. PCT levels are not elevated in viral infections because interferon-gamma, which is produced in viral infections, inhibits PCT production [4]. Therefore, a cut-off value of 0.5 ng/mL has been established as a biomarker for the diagnosis of bacterial infections in adults and children [12,13]. As a biomarker for neonatal bacterial infections, Park et al. have reported that when a cut-off value of 0.5 ng/mL for PCT was used for diagnosing neonatal late-onset bacterial infections, the sensitivity and specificity were 88% and 58%, respectively [14], which are insufficient for clinical applicability. The cut-off value of PCT for the diagnosis of early-onset neonatal bacterial infections has not been determined. This could be because the serum PCT level has been found to show spontaneous variability in neonates during an early age after birth owing to distress during delivery and the different levels between term and preterm infants [15,16,17,18,19,20]. Moreover, the serum PCT level is elevated in the presence of hypoxia, respiratory disorders, asphyxia, intracranial hemorrhage, and hemodynamic abnormalities [21,22]. These are some of the reasons why cut-off values for PCT have not been established for neonates.

To solve these problems, some reference curves of serum PCT levels during early age for neonates have been reported [17,18,19,20]. Fukuzumi et al. investigated 1267 serum samples of 283 Japanese neonates aged 0–5 days and created serum PCT reference curves for preterm, late preterm, and term infants [17]. Turner et al. studied 283 non-infected blood samples from 100 preterm infants aged 0–4 days delivered at a gestational age of ≤36 weeks and created two serum PCT reference curves for preterm infants delivered at a gestational age of 24–30 and 31–36 weeks [18]. Chiesa et al. (2011) created two serum PCT reference curves from 0 to 5 days of age for preterm infants delivered at a gestational age of 30–36 weeks and term infants delivered at a gestational age of 37–39 weeks based on data obtained from 200 preterm infants and 221 term infants, respectively [19]. Chiesa et al. (1998) also created a serum PCT reference curve from 0 to 48 h of age for term infants based on the data of 124 blood samples obtained from 83 healthy term infants [20]. To date, no clinical studies have been conducted to compare the accuracy and precision of these serum PCT reference curves.

Therefore, first, we aimed to validate the diagnostic accuracy of these PCT reference curves for the detection of early-onset neonatal bacterial infections. Second, we aimed to determine which biomarkers among a cut-off value of the 95th percentile in the serum PCT reference curve, WBC count, and CRP and IgM levels, alone or in combination, have the highest diagnostic accuracy for detecting early-onset neonatal bacterial infections.

## 2. Patients and Methods

### 2.1. Study Design and Subjects

We conducted a retrospective cohort study among 39 neonates admitted to the neonatal intensive care unit of Nihon University Itabashi Hospital owing to a suspected bacterial infection that developed within 72 h after birth between 2014 and 2018. The study subjects were divided into two groups: 16 preterm infants delivered at a gestational age of <37 weeks and 23 term infants delivered at a gestational age of ≥37 weeks. Each category was divided into two subgroups depending on the diagnosis of an early-onset bacterial infection: confirmed infection and non-infection. On admission, all patients underwent blood examinations, including evaluations of WBC count, CRP level, IgM level, and PCT level, and cultures of blood, nasal swab, urine, gastric fluid, and stool samples. Cerebrospinal fluid cultures were also obtained for patients with suspected meningitis. The study protocol was approved by the ethics committee for clinical research of Nihon University Itabashi Hospital (approval no. RK-190910-8, date 12 September 2019). Formal written informed consent was not required owing to the retrospective nature of the study, which used anonymized data generated from our regular practice. This study project was open to the public in Japanese by assessing our website (http://www.med.nihon-u.ac.jp/hospital/itabashi/cr/index.html).

### 2.2. Suspected or Confirmed Diagnosis of an Early-Onset Bacterial Infection

Early-onset bacterial infection was suspected upon identifying one or more of the following clinical findings and treatments in the mother: elevated serum CRP level, group B *Streptococcus* (GBS) colonization in vaginal secretions, premature rupture of membranes (PROM), and intrapartum antibiotic use, and/or when attending neonatologists identified one or more of the following clinical symptoms in the newborn: fever, hypothermia, temperature instability, apnea, bradycardia, tachycardia, reduced urine output, respiratory distress, increased oxygen requirement, rash, feeding intolerance, abdominal distension, poor sucking, irritability, lethargy, or hypotonia [23,24]. The final confirmed diagnosis of early-onset bacterial infection was made as culture-proven sepsis or clinical sepsis based on any positive culture results (Section 2.3.).

### 2.3. Definitions of Culture-Proven Sepsis, Clinical Sepsis, and Systemic Inflammatory Response Syndrome

In addition to the decision made based on clinical symptoms or findings and the clinical course with antibiotic treatments provided by attending neonatologists, culture-proven sepsis was diagnosed when any pathogen was detected via blood culture [3]. Clinical sepsis was diagnosed when any pathogen was detected via culture of the nasopharyngeal swab, gastric aspirate, stool, or urine samples [24]. A positive culture result for coagulase-negative *Staphylococci* was not indicative of culture-proven sepsis or clinical sepsis. Systemic inflammatory response syndrome (SIRS) was defined as the presence of two or more of the following conditions using the International Pediatric Sepsis Consensus Conference criteria for neonates aged 0 days to 1 week: hyperthermia or hypothermia, bradycardia or tachycardia, tachypnea, and an increased or decreased leukocyte count or more than 10% immature leukocytes in the peripheral blood [25].

### 2.4. Methods of Measuring Each Biomarker and Culture

Serum PCT, CRP, and IgM levels were measured using Lumipulse Presto Brahms PCT kits (FUJIREBIO Inc., Tokyo, Japan), LZ Test EIKEN CRP-HG (Eiken Chemical Co., Ltd., Tokyo, Japan), and N-assay TIA IgM-SH Nittobo (Nittobo Medical Co., Ltd., Tokyo, Japan), respectively, as recommended by the manufacturers. WBC counts and their differential counts were measured with an automated hematology analyzer (XN-9100; Sysmex Co., Ltd., Kobe, Japan).

For culture, blood, nasal swab, and stool samples were collected using BD BACTEC™ Peds Plus™ Media (Becton, Dickinson and Company, Franklin Lakes, NJ, USA), Transystem™ 116C (COPAN Diagnostics Inc., Murrieta, CA, USA), and Transystem™ 108C (COPAN Diagnostics Inc., Murrieta, CA, USA), respectively. Urine, gastric aspirate, and cerebrospinal fluid samples were collected in sterile spitz (Eiken Chemical Co., Ltd., Tokyo, Japan). Cultures were performed using blood and chocolate agar to identify the organisms. Positive blood culture results were detected using the BD BACTEC™ FX System (Becton, Dickinson and Company, Franklin Lakes, NJ, USA) as recommended by the manufacturer.

### 2.5. Study Methods

#### 2.5.1. Clinical Characteristics of Each Group and the Causative Organisms of Confirmed Infections

Maternal clinical characteristics, including complications of pregnancy (gestational diabetes and hypertensive disorders of pregnancy), delivery mode, GBS colonization status, PROM, and intrapartum antimicrobial treatment, were compared between the confirmed and uninfected groups. We compared the clinical characteristics of the newborns, including birth weight; gestational age at birth; sex; Apgar score; umbilical cord blood power of hydrogen; severe neonatal asphyxia (Apgar score ≤3 at 1 min post-delivery); respiratory disorders (such as respiratory distress syndrome, transient tachypnea of the newborn, meconium aspiration syndrome, and air leak syndrome); intracranial hemorrhage; patent ductus arteriosus that required indomethacin or surgical treatment; SIRS; use of a ventilator, catecholamine, or direct hemoperfusion with polymyxin B-immobilized fiber column; age at measurement of infection biomarkers; and PCT, CRP, WBC, and IgM values between the groups. In the confirmed infection group, the detected pathogens, samples yielding a positive culture, and clinical diagnosis were studied.

#### 2.5.2. Validation of Serum PCT Reference Curves

Serum PCT reference curves were obtained from four previous reports [17,18,19,20]: (a) Fukuzumi et al., (b) Turner et al., and (c) Chiesa et al. (2011) for preterm infants, and (d) Fukuzumi et al., (e) Chiesa et al. (1998), and (f) Chiesa et al. (2011) for term infants. For preterm infants in the present study, the reference curve for preterm infants delivered at a gestational age of <34 weeks published by Fukuzumi et al., was used as (a) and the reference curve for neonates delivered at a gestational age of 31–36 weeks published by Turner et al. was used as (b) [17,18]. The cut-off serum PCT value considered indicative of bacterial infection was one over the 95th percentile for each reference curve [17]. Since the reference curve (e) was only available for up to 48 h of age [20], two infants who were admitted and underwent blood sample collection from 48 to 72 h of age were excluded.

Serum PCT values on age after birth, when measured, were plotted on the aforementioned reference curves, and the number of infants whose serum PCT values exceeded the 95th percentile on each reference curve was counted in each group and analyzed for diagnostic accuracy.

#### 2.5.3. Most Useful Biomarker for Early-Onset Neonatal Bacterial Infection

The cut-off values of the biomarkers that were commonly used for the diagnosis of neonatal bacterial infections were CRP level ≥1.0 mg/dL, WBC count ≥25,000/μL or <5000/μL, IgM level ≥20 mg/dL [3], and serum PCT level over the 95th percentile on the reference curve with the highest Youden index (Section 2.5.2). The diagnostic accuracy of each biomarker alone or in combination for early-onset bacterial infections was analyzed.

#### 2.5.4. Statistical Analysis

Fischer’s exact test or the Mann–Whitney *U* test was used to compare the datasets of the two groups, as appropriate. The diagnostic accuracy was determined using the sensitivity, specificity, accuracy, and the Youden index on the receiver operating characteristic curve analysis. The Youden index is the point farthest from the boundary delineating the area under the curve and represents the (sensitivity + specificity – 1) value [26]. All statistical analyses were performed using JMP version 14 (SAS Institute Inc., Tokyo, Japan). *p*-values <0.05 were considered significant.

## 3. Results

### 3.1. Patient Characteristics and Pathogens Detected in the Confirmed Infection Group

#### 3.1.1. Preterm Infants

Of the 16 patients suspected of having an early-onset bacterial infection, 6 were in the confirmed infection group and 10 in the non-infection group. There was no significant difference in the maternal clinical characteristics between the confirmed and non-infection groups. Regarding the neonatal clinical characteristics, the frequency of SIRS was significantly higher in the confirmed infection group than in the non-infection group, as expected. For other clinical backgrounds, although no significant difference was found in the age of measurement of infection biomarkers between the two groups, PCT and CRP levels were significantly higher, and WBC count was significantly lower in the confirmed infection group than in the non-infection group. There was no significant difference in IgM levels between the two groups (Table 1). The distributions of detected pathogens and samples used for culture in the confirmed infection group are shown in Table 2. Two infants had culture-proven sepsis, and four had clinical sepsis.

#### 3.1.2. Term Infants

Of the 23 patients suspected of having an early-onset bacterial infection, 6 were in the confirmed infection group and 17 in the non-infection group. There was no significant difference in maternal and neonatal clinical characteristics between the two groups. No difference was found in the age at the measurement of infection biomarkers, and there were no significant differences between the two groups in each biomarker (Table 3). The distributions of detected pathogens and the samples used for culture in the confirmed infection group are shown in Table 4. One infant had culture-proven sepsis and five had clinical sepsis.

### 3.2. Validation of Serum PCT Reference Curves

**Figure 1:**
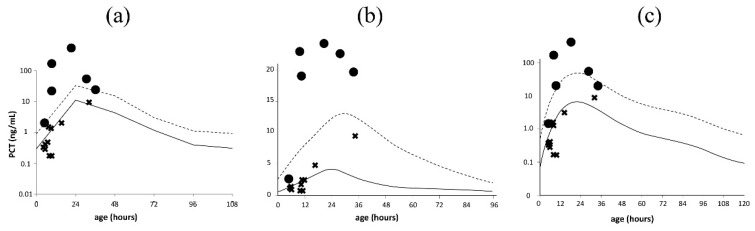
Serum PCT levels of preterm infants in the confirmed and non-infection groups. Serum PCT reference curves (**a**–**c**) are the serum PCT reference curves published by Fukuzumi et al. [17], Turner et al. [18], and Chiesa et al. (2011) [19]. Solid and dotted lines indicate the 50th and 95th percentile lines, respectively. ● represents a PCT value for an infant with confirmed infection; × represents a PCT value for a non-infected infant. PCT, procalcitonin.

**Figure 2:**
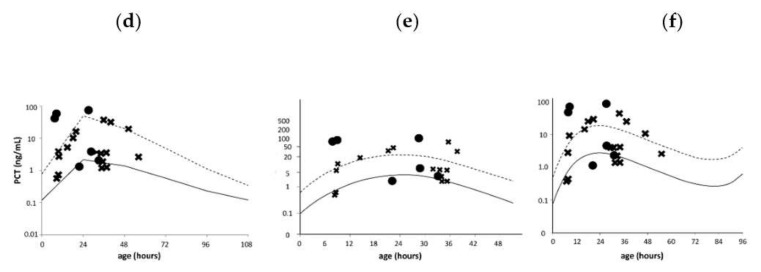
Serum PCT levels of term infants in the confirmed and non-infection groups. Serum PCT reference curves (**d**–**f**) are the serum PCT reference curves published by Fukuzumi et al. [17], Chiesa et al. (1998) [20] and Chiesa et al. (2011) [19]. Since the reference curve (**e**) was only available for neonates aged up to 48 h [20], two infants who were admitted and underwent blood sample collection from 48 to 72 h of age were excluded. Solid and dotted lines indicate the 50th and 95th percentile lines, respectively. ● represents a PCT value for an infant with confirmed infection; × represents a PCT value for a non-infected infant. PCT, procalcitonin.

Because serum PCT levels are dependent on the postnatal age, Figure 1 and Figure 2 represent the 50th and 95th percentile lines of serum PCT in preterm and term newborn according to their postnatal age, respectively [17,18,19,20]. The serum PCT levels of infants in the confirmed and non-infection groups were plotted on each serum PCT reference curve (Figure 1 and Figure 2). The sensitivity, specificity, accuracy, and Youden index of each reference curve for the detection of confirmed infection were calculated (Table 5). The reference curve (a) for preterm infants and (d) for term infants had the highest Youden indexes.

### 3.3. Useful Biomarkers for Detection of Early-Onset Neonatal Bacterial Infections

The serum PCT reference curves (a) and (d) in preterm and term infants, respectively, were used based on the results shown in Table 5. Table 6 shows the results of sensitivity, specificity, accuracy, and Youden index for each biomarker, either alone or in combination, for the detection of confirmed infection. Among preterm infants, the Youden index for PCT alone was 1.000. Among term infants, a combination of PCT, CRP, and WBC and/or IgM yielded a Youden index of 1.000.

## 4. Discussion

Since serum PCT levels become transiently elevated early after birth, the cut-off value for early-onset bacterial infections cannot be determined and its reference curves have been reported instead [17,18,19,20]. In the present study, for the first time, we analyzed the diagnostic accuracy of these three reference curves for the detection of early-onset neonatal bacterial infections among preterm and term infants. As a result, the serum PCT reference curves published by Fukuzumi et al. [17] had the highest diagnostic accuracy than the serum PCT reference curves published by others among preterm and term infants. Furthermore, we found that the serum PCT level over the 95th percentile on the reference curve had sufficient diagnostic accuracy for detecting early-onset bacterial infections among preterm infants, whereas a combination of PCT and CRP levels with WBC count and/or IgM level provided sufficient diagnostic accuracy among term infants.

In children, SIRS is generally diagnosed as sepsis [25]. However, the definitions of SIRS correlated insufficiently to the diagnosis of culture-proven and clinical early-onset sepsis among neonates [27,28]. In the present study, therefore, patients with SIRS were not always included among those with a confirmed diagnosis of culture-proven and clinical early-onset sepsis. Furthermore, clinical sepsis is generally diagnosed using laboratory parameters, such as the WBC count, immature to total neutrophils ratio, and CRP level, in addition to the aforementioned clinical symptoms [27] (Section 2.2.). As the present study validated laboratory biomarkers, clinical sepsis considered that non-contaminating pathogens were detected in culture from at least one sample other than blood, which is a diagnostic criterion for clinical sepsis according to Krueger et al. [24]. In other words, all infants with confirmed early-onset bacterial infections in the present study were diagnosed based on culture results.

Of the reference curves for preterm infants, (a) was clinically useful because of its high diagnostic accuracy. As reference curve (a) had a lower PCT value of the 95th percentile at 6 h of age than (b) and (c), all infants with confirmed infection were detected. Therefore, a PCT value of approximately 1.0 ng/mL at the 95th percentile of the PCT reference curve within 6 h of age would be appropriate. The sensitivity of reference curve (c) was lower than those of the others, but this may have been influenced by the higher 95th percentile value from 12 to 36 h of age than that of others. All reference curves (a) to (c) had a specificity of 1.00. Because preterm infants typically develop respiratory disorders, such as respiratory distress syndrome and transient tachypnea of the newborn even in the non-infection group, the reference curve did not seem to have a significant impact on the respiratory disorders, which are characterized by an elevated serum PCT level.

Among the reference curves for term infants, (d) had the highest Youden index. However, its diagnostic accuracy regarding sensitivity, specificity, and accuracy was limited because infants in the non-infection group had a high PCT level due to other clinical factors, such as respiratory disorders, asphyxia, intracranial hemorrhage, and hemodynamic abnormalities [21,22]. Although there was no difference in sensitivity among the reference curves, we believe that the higher specificity of reference curve (d) was due to the higher PCT value at the 95th percentile at 24 h of age than in the other two. Yan et al. have reported that the causative pathogens and the site of infection showed differences in the degree of PCT elevation, and Gram-negative bacteria were more likely to result in high PCT levels among patients with positive blood culture results [29]. In the present study, the small number of patients with positive blood culture results and the fact that *Streptococcus* was the most common causative organism may have affected the diagnostic accuracy. 

Among preterm infants, using the 95th percentile value as the cut-off value in the PCT reference curve (a), it was possible to determine early-onset bacterial infections. We showed that the CRP level, WBC count, and IgM level, other than the PCT level, do not provide sufficient diagnostic accuracy, either alone or in combination, for the detection of early-onset bacterial infections. CRP levels induced by infection are significantly lower in preterm infants than in term infants, and its sensitivity for the diagnosis of neonatal sepsis is low [30,31]. Hornik et al. found that a WBC count <5000/µL is associated with an increased risk of bacterial infection and reported a lower sensitivity for the detection of early-onset neonatal sepsis [32]. In the present study, the median WBC count was lower in the confirmed infection group than in the non-infection group; only one infant had a WBC count < 5000/µL. Krishna et al. found higher IgM levels among infants with early-onset neonatal sepsis and characterized the IgM level as a biomarker with high specificity [7]. In the present study, even in the confirmed infection group, the median IgM level was <10 mg/dL, which was not necessarily high, but the specificity was as high as 0.900. In contrast, among term infants, a cut-off value at the 95th percentile in the reference curve (d) did not provide sufficient diagnostic accuracy. Among term infants, PCT and CRP levels and WBC count are also elevated in the presence of respiratory disorders, neonatal asphyxia, intracranial hemorrhage, and hemodynamic abnormalities [21,22,33]; therefore, we showed in the present study that a combination of PCT level, CRP level, WBC count, and/or IgM level could detect early-onset neonatal bacterial infections.

Of the 39 infants enrolled in our study, 27 were free of bacterial infection. However, 26 infants (9 preterm and 17 term) underwent treatment with antimicrobial agents. Based on our results, for newborns suspected of having early-onset bacterial infections, using a cut-off value of the 95th percentile in the reference curve for preterm infants or a combination of PCT level, CRP level, WBC count, and/or IgM level for term infants would lead to the appropriate use of antimicrobial drugs and prevent antimicrobial resistance [22].

Our study had several limitations. First, we assessed a limited number of patients at a single center. However, the incidence of early-onset culture-proven sepsis in Japan is currently very low (approximately 0.1% even in 2006–2008) [3]. In this context, it was valuable to evaluate for the first time the diagnostic accuracy of several serum PCT reference curves that have been reported so far [17,18,19,20]. This is also the first report to clarify the diagnostic accuracy of serum PCT reference curves for early-onset bacterial infection. Second, it was not possible to study pathological chorioamnionitis because some enrolled infants were delivered elsewhere and transferred to our hospital. Further prospective studies using a large number of infants are needed to draw a final conclusion. In addition, the results of the present study suggest the need to conduct clinical studies to determine whether our criteria will result in the appropriate use of antimicrobial agents and serve as a measure against antimicrobial resistance.

## 5. Conclusions

Among previously published serum PCT reference curves, the serum PCT reference curve published by Fukuzumi et al. had the highest diagnostic accuracy. For preterm infants, the PCT level alone with a cut-off value over the 95th percentile in the serum PCT reference curve, and for term infants, the PCT level, and CRP level in combination with WBC count and/or IgM level should be used for the detection of early-onset bacterial infection.

## Figures and Tables

**Table 1:** Distribution of clinical characteristics of preterm infants.

	Confirmed Infection *n* = 6	Non-Infection *n* = 10	*p*-Value
A. Maternal characteristics			
Gestational diabetes **	0 (0)	0 (0)	-
Hypertensive disorders of pregnancy **	0 (0)	1 (10)	1.00
Delivery mode **			0.52
Vaginal delivery	2 (33)	1 (10)	
Cesarean section	4 (67)	9 (90)	
GBS colonization **	3 (50)	2 (20)	0.30
PROM **	4 (67)	3 (30)	0.30
Intrapartum antimicrobial treatment **	5 (83)	9 (90)	1.00
B. Neonatal characteristics			
Birth weight, g *	1697 (538–2226)	1551 (549–2422)	0.96
Gestational age, weeks *	31.5 (23.9–35.6)	32.0 (24.0–36.7)	0.83
Male sex **	5 (83)	5 (50)	0.31
Apgar score			
at 1 min *	4.0 (1–8)	6.5 (1–9)	0.47
at 5 min *	6.5 (4–9)	7.5 (1–9)	0.34
Umbilical cord blood pH *	7.235 (7.109–7.360)	7.304 (6.888–7.427)	0.30
Severe neonatal asphyxia **	3 (50)	3 (30)	0.61
Respiratory disorder **	6 (100)	9 (90)	1.00
Intracranial hemorrhage **	2 (33)	0 (0)	0.13
Patent ductus arteriosus that required indomethacin or surgical treatment **	2 (33)	5 (50)	0.63
SIRS **	6 (100)	0 (0)	<0.001
Antimicrobial treatment **	6 (100)	9 (90)	1.00
ABPC and AMK	3 (50)	8 (80)	
ABPC and CTX	2 (33)	0 (0)	
ABPC and CMZ	0 (0)	1 (10)	
ABPC	1 (17)	0 (0)	
Use of ventilator **	6 (100)	6 (60)	0.23
Use of catecholamine **	6 (100)	6 (60)	0.23
Use of PMX-DHP **	2 (33)	0 (0)	0.13
Death **	1 (17)	0 (0)	0.38
Age at measurement of biomarkers, days *	0.5 (0–2)	0 (0–2)	0.13
PCT, ng/mL *	43.7 (1.45–200)	0.6 (0.25–8.25)	0.004
CRP, mg/dL *	1.42 (0.1–5.82)	0.1 (0.1–5.67)	0.01
WBC, /µL *	6350 (2800–9900)	9900 (7600–18,000)	0.01
IgM, mg/dL *	7.5 (2–14)	8.5 (4–25)	0.51

* Data were presented as the median (range) and compared by using Mann–Whitney *U* test. ** Data were presented as number (percentage) and compared by using Fischer’s exact test. AMK, amikacin; ABPC, ampicillin; CMZ, cefmetazole; CTX, cefotaxime; CRP, C-reactive protein; PMX-DHP, direct hemoperfusion with polymyxin B-immobilized fiber column; GBS, group B *Streptococcus*; IgM, immunoglobulin M; pH, power of hydrogen; PROM, premature rupture of membranes; PCT, procalcitonin; SIRS, systemic inflammatory response syndrome; WBC, white blood cell.

**Table 2:** Pathogens detected and samples used for culture among preterm infants in the confirmed diagnosis group.

No.	Pathogens Detected	Samples	Clinical Diagnosis
1	*Streptococcus agalactiae*	Nasopharyngeal swab, stool, and gastric aspirate	Clinical sepsis
2	*Escherichia coli*	Gastric aspirate	Clinical sepsis
3	*Bacillus cereus*	Blood	Culture-proven sepsis
4	*Enterococcus faecalis*	Nasopharyngeal swab, stool, and gastric aspirate	Clinical sepsis
5	*Enterococcus faecalis*	Blood, nasopharyngeal swab, and gastric aspirate	Culture-proven sepsis
6	*Enterococcus faecalis, Enterobacter aerogenes, Klebsiella pneumoniae*	Urine and gastric aspirate	Clinical sepsis

**Table 3:** Distribution of clinical characteristics among term infants

	Confirmed Infection *n* = 6	Non-Infection *n* = 17	*p*-Value
A. Maternal characteristics			
Gestational diabetes **	0 (0)	0 (0)	-
Hypertensive disorders of pregnancy **	0 (0)	0 (0)	-
Delivery mode **			0.61
Vaginal delivery	5 (83)	13 (76)	
Cesarean section	1 (17)	4 (24)	
GBS colonization **	2 (33)	2 (12)	0.27
PROM **	3 (50)	7 (41)	1.00
Intrapartum antimicrobial treatment **	3 (50)	8 (47)	1.00
B. Neonatal characteristics			
Birth weight, g *	2959 (2485–3572)	3165 (2424–3732)	0.38
Gestational age, weeks *	39.5 (37.1–41.3)	40.0 (37.7–40.9)	0.79
Male sex **	4 (67)	8 (47)	0.41
Apgar score			
at 1 min *	9.0 (7–9)	8.0 (1–9)	0.27
at 5 min *	9.0 (8–10)	9.0 (5–10)	0.82
Umbilical cord blood pH *	7.340 (7.254–7.400)	7.306 (7.100–7.390)	0.31
Severe neonatal asphyxia **	0 (0)	2 (12)	1.00
Respiratory disorder **	3 (50)	6 (35)	0.64
Intracranial hemorrhage **	0 (0)	3 (18)	0.54
SIRS **	1 (17)	0 (0)	0.26
Antimicrobial treatment **	6 (100)	17 (100)	-
ABPC and AMK	5 (83)	17 (100)	
ABPC and CTX	1 (17)	0 (0)	
Use of ventilator **	1 (17)	0 (0)	0.26
Use of catecholamine **	1 (17)	1 (6)	0.46
Use of PMX-DHP **	1 (17)	0 (0)	0.26
Death **	0 (0)	0 (0)	-
Age at measurement of biomarkers, days *	0.5 (0–1)	1.0 (0–3)	0.76
PCT, ng/mL *	40.4 (1.26–94.1)	6.37 (0.79–65.6)	0.25
CRP, mg/dL *	5.15 (0.65–10.31)	4.52 (1.02–14.62)	0.70
WBC, /µL *	20,650 (1900–36,300)	18,100 (11,300–42,500)	1.00
IgM, mg/dL *	7.5 (5–11)	13 (6–34)	0.05

* Data were presented as the median (range) and compared by using Mann–Whitney *U* test. ** Data were presented as number (percentage) and compared by using Fischer’s exact test. AMK, amikacin; ABPC, ampicillin; CTX, cefotaxime; CRP, C-reactive protein; PMX-DHP, direct hemoperfusion with polymyxin B-immobilized fiber column; GBS, group B *Streptococcus*; IgM, immunoglobulin M; pH, power of hydrogen; PROM, premature rupture of membranes; PCT, procalcitonin; SIRS, systemic inflammatory response syndrome; WBC, white blood cell.

**Table 4:** Pathogens detected and samples used for culture among term infants in the confirmed diagnosis group.

No.	Detected Pathogens	Samples	Clinical Diagnosis
1	*Streptococcus agalactiae*	Stool	Clinical sepsis
2	*Streptococcus agalactiae, Escherichia coli*	Nasopharyngeal swab, stool, and gastric aspirate	Clinical sepsis
3	*Streptococcus agalactiae, Escherichia coli*	Nasopharyngeal swab, stool, and gastric aspirate	Clinical sepsis
4	*Streptococcus agalactiae, Escherichia coli*	Urine	Clinical sepsis
5	*Escherichia coli*	Nasopharyngeal swab, stool, and gastric aspirate	Clinical sepsis
6	*Streptococcus pyogenes*	Blood and nasopharyngeal swab	Culture-proven sepsis

**Table 5:** Comparisons among serum PCT reference curves.

Reference Curve	Sensitivity	Specificity	Accuracy	Youden Index
A. Preterm infants				
(a)	1.000	1.000	1.000	1.000
(b)	0.833	1.000	1.000	0.833
(c)	0.667	1.000	1.000	0.667
B. Term infants				
(d)	0.500	0.824	0.739	0.324
(e)	0.500	0.667	0.619	0.167
(f)	0.500	0.588	0.565	0.088

PCT, procalcitonin.

**Table 6:** Comparisons among the four biomarkers, alone or in combination.

Reference Curve	Sensitivity	Specificity	Accuracy	Youden Index
A. Preterm infants				
PCT	1.000	1.000	1.000	1.000
CRP	0.667	0.800	0.750	0.467
WBC	0.167	1.000	0.688	0.167
IgM	0.000	0.900	0.563	−0.100
PCT and/or CRP	1.000	1.000	1.000	1.000
PCT and/or WBC	1.000	1.000	1.000	1.000
PCT and/or IgM	1.000	1.000	1.000	1.000
CRP and/or WBC	0.667	1.000	0.875	0.667
CRP and/or IgM	0.667	1.000	0.875	0.667
WBC and/or IgM	0.167	1.000	0.688	0.167
PCT, CRP, and/or WBC	1.000	1.000	1.000	1.000
PCT, CRP, and/or IgM	1.000	1.000	1.000	1.000
PCT, WBC, and/or IgM	1.000	1.000	1.000	1.000
CRP, WBC, and/or IgM	0.667	1.000	0.875	0.667
PCT, CRP, WBC, and/or IgM	1.000	1.000	1.000	1.000
B. Term infants				
PCT	0.500	0.824	0.739	0.324
CRP	0.833	0.000	0.217	−0.167
WBC	0.500	0.824	0.739	0.324
IgM	0.000	0.765	0.565	−0.235
PCT and/or CRP	1.000	0.824	0.870	0.824
PCT and/or WBC	0.667	1.000	0.913	0.667
PCT and/or IgM	0.500	1.000	0.870	0.500
CRP and/or WBC	1.000	0.824	0.870	0.824
CRP and/or IgM	0.833	0.765	0.783	0.598
WBC and/or IgM	0.500	0.882	0.783	0.382
PCT, CRP, and/or WBC	1.000	1.000	1.000	1.000
PCT, CRP, and/or IgM	1.000	1.000	1.000	1.000
PCT, WBC, and/or IgM	0.667	1.000	0.913	0.667
CRP, WBC, and/or IgM	1.000	0.882	0.913	0.882
PCT, CRP, WBC, and/or IgM	1.000	1.000	1.000	1.000

CRP, C-reactive protein; WBC, white blood cell; IgM, immunoglobulin M; PCT, procalcitonin.

## Abbreviations

WBC	White blood cell
CRP	C-reactive protein
IgM	Immunoglobulin M
PCT	Procalcitonin
GBS	Group B *Streptococcus*
PROM	Premature rupture of membranes
SIRS	Systemic inflammatory response syndrome
