# Correction: Go et al. Diagnostic Accuracy of Biomarkers for Early-Onset Neonatal Bacterial Infections: Evaluation of Serum Procalcitonin Reference Curves. *Diagnostics* 2020, *10*, 839

**DOI:** 10.3390/diagnostics12102277

**Published:** 2022-09-21

**Authors:** Hidetoshi Go, Nobuhiko Nagano, Daichi Katayama, Takuya Akimoto, Takayuki Imaizumi, Ryoji Aoki, Midori Hijikata, Ayako Seimiya, Ryota Kato, Aya Okahashi, Ichiro Morioka

**Affiliations:** Department of Pediatrics and Child Health, Nihon University School of Medicine, Tokyo 173-8610, Japan

In the original publication [1], there were some mistakes. The symbols in Figure 2 were (d), (e), (f), not (a), (b), (c). Accordingly, in the legend of Figure 2, (a–c) was corrected to (d–f) and (b) was corrected to (e). In addition, the reference Chiesa et al. (1988) [20] was corrected to Chiesa et al. (1998) [20].

The authors apologize for any inconvenience caused and state that the scientific conclusions are unaffected. This correction was approved by the Academic Editor. The original publication has also been updated.

## Figures and Tables

**Figure 2:**
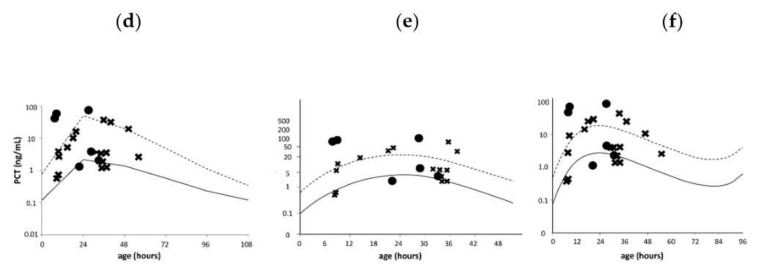
Serum PCT levels of term infants in the confirmed and non-infection groups. Serum PCT reference curves (**d**–**f**) are the serum PCT reference curves published by Fukuzumi et al. [17], Chiesa et al. (1998) [20] and Chiesa et al. (2011) [19]. Since the reference curve (**e**) was only available for neonates aged up to 48 h [20], two infants who were admitted and underwent blood sample collection from 48 to 72 h of age were excluded. Solid and dotted lines indicate the 50th and 95th percentile lines, respectively. ● represents a PCT value for an infant with confirmed infection; **×** represents a PCT value for a non-infected infant. PCT, procalcitonin.

## References

[1] Go H., Nagano N., Katayama D., Akimoto T., Imaizumi T., Aoki R., Hijikata M., Seimiya A., Kato R., Okahashi A. (2020). Diagnostic Accuracy of Biomarkers for Early-onset Neonatal Bacterial Infections: Evaluation of Serum Procalcitonin Reference Curves. Diagnostics.

